# Low cardiovascular risks in the middle aged males and females excreting greater 24-hour urinary taurine and magnesium in 41 WHO-CARDIAC study populations in the world

**DOI:** 10.1186/1423-0127-17-S1-S21

**Published:** 2010-08-24

**Authors:** Yukio Yamori, Takashi Taguchi, Hideki Mori, Mari Mori

**Affiliations:** 1 Mukogawa Women’s University Institute for World Health Development, Nishinomiya, Hyogo, 6638143, Japan

## Abstract

**Background:**

Since taurine (T) administration was proven to decrease blood pressure (BP) and stroke mortality in stroke-prone spontaneously hypertension rates (SHRSP) in the 1980’s and our WHO-coordinated CARDIAC (Cardiovascular Diseases and Alimentary Comparison) Study demonstrated that among 5 diet-related factors, namely total cholesterol (T-Cho), body mass index (BMI), sodium (Na), magnesium (M), and T to creatinine (Cr) ratio in 24-hour urine (24U), both T/Cr and M/Cr were inversely related to coronary heart disease mortalities in males and females and T/Cr was inversely related to stroke mortalities in males and females. We further analyzed the associations of individual T/Cr and M/Cr levels to cardiovascular risks in the present study.

**Method:**

From WHO-CARDIAC Study populations, 61 populations of 25 countries in the world, Japanese populations with obviously higher 24U T excretion because of their common fish eating custom and the other populations in which both data of T and M were not available were excluded and the data of 3960 individuals from 41 WHO-CARDIAC Study populations were used for the following analyses.

**Results:**

The means of 24U T/Cr and M/Cr ratios in total individual data were 639.4 and 82.8, respectively. The average of BMI, systolic and diastolic blood pressure (SBP, DBP), T-Cho and atherogenic index (AI) in the individuals with more than the means of T/Cr or M/Cr were significantly lower than those of individuals with less than the means. The CARDIAC Study participants were divided into the following 4 groups by these means: A (T/Cr and M/Cr ≧ mean), B (T/Cr ≧ mean, M/Cr < mean), C (T/Cr < mean, M/Cr ≧ mean), D (T/Cr and M/Cr < mean). The group A showed significantly lower values compared with the group D in BMI, SBP, DBP, T-Cho, and AI.

**Conclusions:**

Cardiovascular risks were proven to be highly significantly lower in individuals who were excreting both 24U T and M, more than the averages despite differences in ethnicity and genetic background. Since T and M are biomarkers for seafood, vegetables, soy, nuts, milk, etc., dietary custom to eat these food sources could be recommended for cardiovascular disease prevention.

## Background

WHO-CARDIAC Study is an epidemiological surveillance covering 61 regions in 25 countries that has been executed since 1985 [1,2]. This study confirmed that there was a close relation between cardiovascular diseases (CVD) and dietary customs by analyzing dietary biomarkers of 24-hour urine (24U). The amount of taurine (T) excreted in 24U and the mortality rates of coronary heart diseases (CHD) were inversely correlated significantly by a regional correlation analysis [3]. Moreover, the mortality rates were found significantly lower in the regions where as much T was consumed as in Japan. On the other hand, when the correlation of 24U magnesium (M) excretion and the blood pressure (BP) was analyzed, M showed inverse correlations with both systolic and diastolic BP’s [4]. These investigations suggested that the risk of the CVD differed in different populations and could be influenced greatly by such environmental factors as dietary customs, lifestyles and temperature. The data so far obtained from the population averages of worldly distributed regions showed that T and M intakes might have protective effect on CVD. However, the population averages are influenced greatly by the genetic factor of the inhabitants and the environmental factors of the regions examined. In the present study, the influence on CVD risks of the dietary factors was analyzed by examining the associations of 24U T and M excretions of individual participants with their own CVD risks disregarding their genetic backgrounds, living conditions and genders in the combined CARDIAC Study population samples.

## Methods

The health examinations for CARDIAC Study were carried out for about 200 in the total of males and females aged 48-56-years randomly selected according to CARDIAC Study protocol; height, weight and BP measured by using an automated BP measurement system were recorded, and fasting blood was collected for measuring total serum cholesterol (T-Cho) and HDL-cholesterol (HDL) to calculate atherogenic index (AI: T-Cho/HDL). 24U samples were collected by using aliquot cups to measure biomarkers of dietary intakes of sodium (Na), potassium (K), T, M, and creatinine (Cr). Cr was measured to calculate these dietary markers per body size by an alkaline picrate method and photometric reading. M was measured by a colorimetric method. T was measured by high performance liquid chromatography. The data of 3960 participants in 41 regions who succeeded in collecting 24U were used after excluding the Japanese where T intakes from seafood were extremely high and CHD mortalities were lowest. Using 24U T and M, the mean values of T/Cr ratio (639.4) and the mean of M/Cr ratio (82.8) were calculated, then all participants whose data were available were divided into four groups, group A (both T/Cr and M/Cr ≧ mean), group B (T/Cr ≧ mean, M/Cr < mean), group C (T/Cr < mean, M/Cr ≧ mean) and group D (both T/Cr and M/Cr < mean), and the averages of risks, BMI, SBP, DBP, T-Cho, HDL and AI were compared between groups. Statistical analyses were conducted with SPSS 15.0J for Windows (SPSS Japan Inc., Tokyo, Japan). Between-group differences in BMI, SBP, DBP, T-Cho and AI were tested by the unpaired Student’s t test. Data are expressed as the mean ± SEM. Values of p < 0.05 were considered significant.

## Results

**Figure 1:**
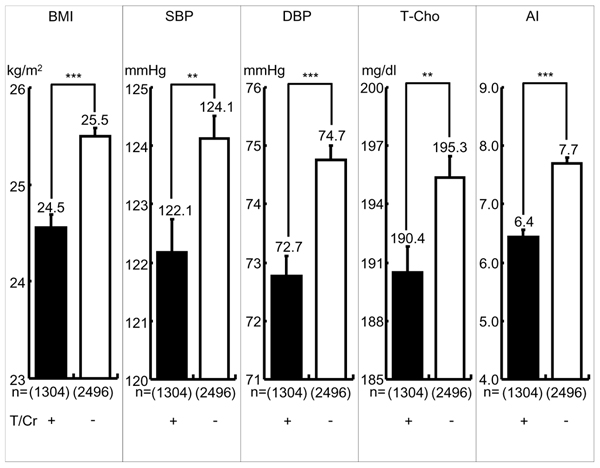
**Cardiovascular Risks in Greater or Lesser 24-hour Urinary Taurine (T) Excreters.** Significant difference: **: p < 0.01, ***: p < 0.001 T/Cr=24-hour urinary taurine (T, micro mol) to creatinine (Cr, g) ratio, Greater T (+) or Lesser T (-) T/Cr: + ≧ mean, or - <mean, mean=639.4 BMI: Body mass index (Body weight (kg)/height (m)^2^), SBP: Systolic blood pressure, DBP: Diastolic blood pressure, T-Cho: Total Cholesterol, AI (Atherogenic index): Total cholesterol/HDL cholesterol, ( ): number

As indicated in Fig. 1, the participants with T/Cr ≧ mean showed significant differences compared with the participants T/Cr < mean in BMI (24.56 ± 0.12 vs. 25.50 ± 0.08 kg/m^2^, p < 0.001), SBP (122.18 ± 0.56 vs. 124.12 ± 0.39 mmHg, p < 0.01), DBP (72.76 ± 0.35 vs. 74.76 ± 0.25 mmHg, p < 0.001), T-Cho (190.48 ± 1.36 vs. 195.37 ± 1.10 mg/dl, p < 0.01) and AI (6.45 ± 0.11 vs. 7.70 ± 0.09, p < 0.001). Between-group differences of BMI, DBP, and AI were highly significant (p < 0.001).

**Figure 2:**
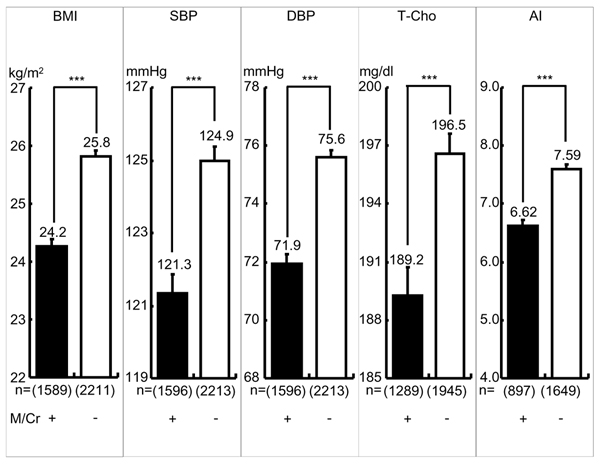
**Cardiovascular Risks in Greater or Lesser 24-hour Urinary Magnesium (M) Excreters.** Significant difference: ***: p < 0.001 M/Cr=24-hour urinary magnesium (M, mg) to creatinine (Cr, g) ratio, greater M (+) or Lesser M (-) Excreters: M/Cr: + ≧ mean, - < mean, mean=82.8 BMI: Body mass index (Body weight (kg)/height (m)^2^), SBP: Systolic blood pressure, DBP: Diastolic blood pressure, T-Cho: Total Cholesterol, AI (Atherogenic index): Total cholesterol/HDL cholesterol, ( )= number

As indicated in Fig. 2, the participants with M/Cr ≧ mean showed all highly significant differences compared with the participants M/Cr < mean in BMI (24.27 ± 0.11 vs. 25.82 ± 25.82 kg/m^2^, p < 0.001), SBP (121.35 ± 0.51 vs. 124.97 ± 0.41 mmHg, p < 0.001), DBP (71.96 ± 0.32 vs. 75.60 ± 0.26 mmHg, p < 0.001), T-Cho (189.25 ± 1.46 vs. 196.57 ± 1.05 mg/dl, p < 0.001) and AI (6.62 ± 0.11 vs. 7.59 ± 0.09, p < 0.001).

**Figure 3:**
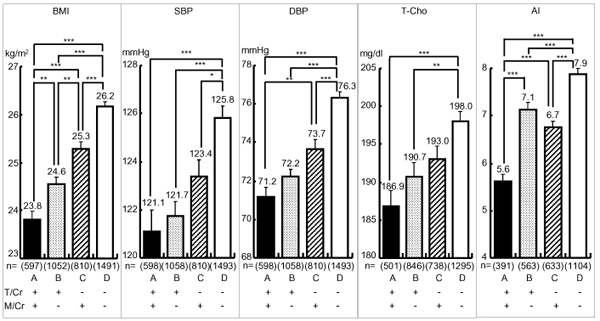
**Cardiovascular Risks in Greater Taurine-Magnesium (T-M) Excreters and Lesser T-M Excreters.** Significant difference: *: p<0.05, **: p<0.01, ***: p<0.001 T/Cr=24-hour urinary taurine (T, micro mol) to creatinine (Cr, g) ratio, M/Cr=24-hour urinary magnesium (M, mg) to Cr, (g) ratio, T/Cr: + ≧ mean, - < mean, mean=639.4, M/Cr: + ≧ mean, - < mean, mean=82.8 BMI: Body mass index (Body weight (kg)/height (m)^2^), SBP: Systolic blood pressure, DBP: Diastolic blood pressure, T-Cho: Total Cholesterol, AI (Atherogenic index): Total cholesterol/HDL cholesterol, ( )=number

Fig. 3 indicated the average of cardiovascular risks in 4 groups, A, B, C and D, classified by the means of T/Cr and M/Cr. Group A (T/Cr, M/Cr ≧ means) showed the lowest risks among four groups and showed highly significant differences compared with group D in the mean values of BMI (23.81 ± 0.18 vs. 26.15 ± 0.11kg/m^2^, p < 0.001), SBP (121.12 ± 0.88 vs. 125.81 ± 0.50 mmHg, p < 0.001), DBP (71.16 ± 0.53 vs. 76.35 ± 0.31 mmHg, p < 0.001), T-Cho (186.85 ± 2.02 vs. 197.96 ± 1.29 mg/dl, p < 0.001), AI (5.63 ± 0.14 vs. 7.87 ± 0.11, p < 0.001). In addition, group A showed significantly lower values compared with group B and C in BMI and AI and with group C in DBP.

## Discussion

The present study confirmed our previous CARDIAC data analyses by the structural equation modeling (SEM) which indicated both T/Cr and M/Cr were strongly inversely related to CHD mortalities in males and females and T/Cr was also inversely related to stroke in males and females [4]. Fig. 1 and 2 clearly demonstrate higher intakes of T/Cr or M/Cr (≥mean) have significantly lower all cardiovascular risks, BMI, SBP, DBP, T-Cho and AI. Fig. 3 indicates that in the individuals whose T/Cr and M/Cr are both over the means, all risks, such as BMI, SBP, DBP and AI, are signficantly lower than in those whose T/Cr and M/Cr are both below the means, indicating enough intakes of both T and M should be the best dietary condition for reducing cardiovascular risks and preventing CHD and stroke as previously reported [3][4]. Since the data analyses were done in the present study regardless of genetic and environmental differences, diets to increase T and M intake, thus to increase T/Cr and M/Cr in 24U, appear to beneficially affect cardiovascular physiology so to reduce cardiovascular diseases. As for the mechanisms of how T and M decrease the risks of cardiovascular diseases, numerous experimental and several clinical studies have been reported. For example, SBP and intracellular calcium (Ca) in peripheral lymphocytes from stroke-prone spontaneously hypertensive rats (SHRSP) were significantly higher than in normotensive Wister-Kyoto rats (WKY), and intracellular M in SHRSP was significantly lower than in WKY [5]. Intracellular Ca overload acts as a trigger of cell death by inducing apoptosis and causes arteriosclerosis [6]. T improves the cardiovascular function through a variety of mechanisms including the improvement of lipid profiles, the modulation of intracellular Ca concentration, antioxidant effects and the antagonism of Ang II action [7]. Dietary T supplementation increases mRNA level of cholesterol 7alpha-hydroxylase to lower serum cholesterol in mice and rats [8]. In addition, T prevents high fat diet induced obesity with increased resting energy consumption [9]. M also activates Na-K ATPase that controls intracellular mineral balance and contributes to the homeostasis of electrolytes in the cell [10]. It was experimentally demonstrated that intracellular Ca and Na was decreased by oral M supplementation which therefore lowered blood pressure in SHRSP [11] and hypertensive patients [12]. High M supplementation lowered AI compared to low M diet group in rat [13]. T and M are involved in the homeostasis of intracellular Ca and Na, thus to contribute to the maintenance of cellular structure and function [10,14]. When the vascular wall cells such as medial smooth muscle cells and endotherial cells are physiologically stabilized, peripheral circulation should be maintained well so that vascular lesions causing CHD and stroke are prevented, thus to contribute to a healthy long life. As for *Klotho* gene related to aging, it affects Na-K ATPase and contributes to intracellular Na and Ca as M affects Na-K ATPase. *Klotho* gene is involved in the maintenance of the Ca homeostasis [15], however, the underling mechanisms of intracellular ionic homeostasis related to M and ageing are not yet clear. Anyhow, the present study indicates the intakes of the seafood containing T as well as seaweeds and nuts rich in M are expected to reduce overall risks related to cardiovascular diseases. Therefore, dietary customs that increase the intake of food containing T and M are expected to improve BP and blood lipid profiles and to decrease cardiovascular diseases.

## Conclusions

The world-wide examination of 24U T and M excretions as the marker of dietary T and M intakes of individual participants in the cross sectional CARDIAC health survey proved a highly significant inverse relationship to major cardiovascular risks as BMI, SBP, DBP, T-Cho and AI, indicating combined intakes of T and M are important for CVD prevention.

## List of abbreviations used

AI: atherogenic index, BMI: body mass index, Ca: Calcium, CARDIAC Study: Cardiovascular Diseases and Alimentary Comparison study, CHD: coronary heart diseases, Cr: creatinine, CVD: cardiovascular diseases, DBP: diastolic blood pressure, K: potassium, M: magnesium, Na: sodium, SBP: systolic blood pressure, SHRSP: stroke-prone spontaneously hypertension rates, T: taurine, T-Cho: total cholesterol, WKY: Wister-Kyoto rats, 24U: 24-hour urine

## Competing interests

The authors declare that they have no competing interests.

## Authors’ contributions

Y.Y. designed this study, T.T. contributed to the data analysis and the preparation of this article, M.M. helped epidemiological data analysis and H.M. contributed for carrying out epidemiological surveys.
